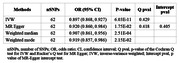# On the Risk of Alzheimer’s Disease in Survivors of Myocardial Infarction: A Mendelian Randomization Study

**DOI:** 10.1002/alz.090073

**Published:** 2025-01-03

**Authors:** Yibeltal Arega Ashebir, Yian Zhang, Yongzhao Shao

**Affiliations:** ^1^ New York University Grossman School of Medicine, New York, NY USA

## Abstract

**Background:**

Myocardial infarction (MI) is a leading cause of death while those surviving MI are still at risk of developing dementias. Alzheimer’s disease (AD) is the leading cause of dementia currently without a cure or an effective prevention even in vulnerable subpopulations. Both AD and MI are common causes of disabilities and deaths in aging populations. It is of interest to investigate AD risk and possible preventive measures for AD among vulnerable aging populations including those surviving MI (survMI).

**Method:**

Employing a two‐sample Mendelian randomization (2SMR) analysis, we assessed the causal relationship between survMI and the risk of AD, utilizing independent AD and MI genome‐wide association study (GWAS) summary datasets. Sensitivity tests, including MR‐Egger intercept test, Cochran Q‐test, and leave‐one‐out analysis, were performed to ensure robustness.

**Result:**

The 2SMR study, utilizing 62 independent single nucleotide polymorphisms (SNPs) as instrumental variables (IVs), revealed a decreased risk of AD associated with survMI (OR = 0.897, p = 6.03E‐11). The MR‐Egger intercept test (p = 0.405) indicated the absence of horizontal pleiotropy, and no evidence of heterogeneity was observed based on the Cochran Q‐test (p = 0.429). Some of the instrumental genetic variants, rs765549, rs11652894, rs12693302, and rs10455872 are linked to LPL, SREBF1, PDE1A, and SLC22A3 genes—targets of medicines and dietary supplements like calcium channel blockers and Omega‐3/fish oil commonly used to enhance MI survival chances.

**Conclusion:**

The 2SMR analysis indicated a significant causal association between survMI and a reduced risk of AD. This suggests that commonly‐used medications, including calcium channel blockers, and supplements like Omega‐3 products, may contribute to lowering the risk of AD. Further studies are essential to validate these findings and explore potential preventive measures for AD using combinations of medications and dietary supplements commonly employed to enhance the probability of surviving MI.